# Supplementary material for: Benchmarking Inverse Statistical Approaches for Protein Structure and Design with Exactly Solvable Models
Source: PLoS Comput Biol. 2016 May 13;12(5):e1004889. doi: 10.1371/journal.pcbi.1004889 (PMC4866778; doi:10.1371/journal.pcbi.1004889)
Supplement: S1 Text — (PDF) [file pcbi.1004889.s001.pdf]

Supporting Information Text S1

Benchmarking inverse statistical approaches for protein structure and design with exactly solvable models

Hugo Jacquin,[1] Amy Gilson,[2] Eugene Shakhnovich,[2] Simona Cocco,[1] and Rémi Monasson[3]

[1]*Laboratory of Statistical Physics, Ecole Normale Supérieure, CNRS, PSL Research University, Sorbonne Universités UPMC, 24 rue Lhomond, 75005 Paris, France*
[2]*Department of Chemistry and Chemical Biology, Harvard University, 12 Oxford Street, Cambridge, MA 02138, USA*
[3]*Laboratory of Theoretical Physics, Ecole Normale Supérieure, CNRS, PSL Research University, Sorbonne Universités UPMC, 24 rue Lhomond, 75005 Paris, France*

I. SEQUENCE SPACE: SAMPLING AND DESIGNABILITY

A. Monte Carlo sampling of the sequence space

Here we describe the Monte Carlo sampling procedure to build the MSA associated with the native structure. As explained in the Method section of the main paper, we want to draw sequences according to the equilibrium measure equal to $P_{\rm nat}(S|A)^\beta$ (up to an irrelevant normalisation factor) [1]. To optimize the sampling of the sequence space we perform multiple mutations at each time step, which greatly reduces the equilibration time and allows us to overcome energy barriers occurring at low temperatures (for inverse temperature $\beta > 10^4$). To this end we draw an integer k from a Poisson distribution of mean 1, and pick out uniformly at random k sites $i_1 < i_2 < ... < i_k$ of the protein. We then mutate uniformly at random each one of the k amino acids into another amino acid.

In Fig. A, we show the average folding probability $P_{\rm nat}$ as a function of the inverse temperature. For $\beta = 10^3$ the average folding probability is about 0.995 for structure S_B and the equilibration time is smaller than 10^3 mutation steps.

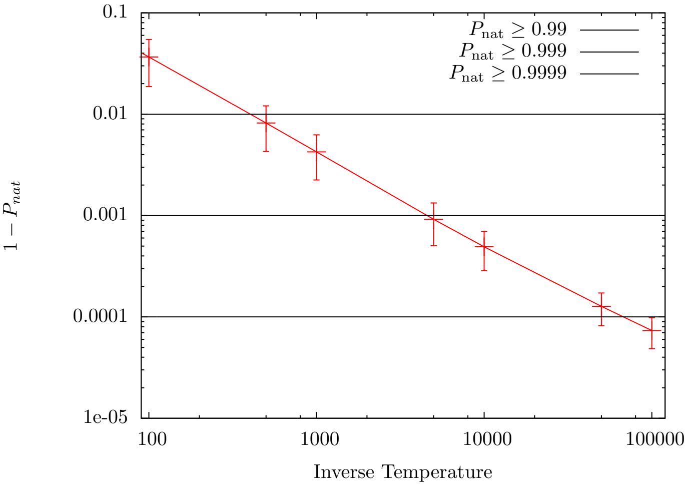

FIG. A: Average probability of misfolding, $1 - P_{\rm nat}$, as a function of the inverse temperature β, for structure S_B. The error bars show two standard deviations from the average value, computed over $M = 5 \cdot 10^4$ sequences.

B. Designability and entropy

An important characteristic of the protein families is their volumes in sequence space, which is captured by the concept of designability. This concept was first introduced by Wingreen and colleagues as the number of sequences that can fold into a given structure [2]. Using two-letter protein alphabet, comprised of hydrophobic and hydrophilic amino acids and the 27-mer lattice model introduced in [3] these authors showed that designabilities of model protein

structures varied broadly from "orphans", i.e. structures that can be encoded by only one sequence, to highly designable structures that can be encoded by multiple sequences. Wingreen and coworkers used a simplified criterion for a sequence to fold into a given (target) conformation – that the target structure is the lowest energy conformation (out of total 103,346 fully compact conformations for the 27-mer model protein). However detailed studies of protein folding using simple lattice and analytical models showed that this condition, albeit necessary, is not sufficient for sequences to encode stable and fast folding proteins [4, 5]. Rather, a large energy gap between the lowest energy (native) conformation and the lowest energy in the set of structurally dissimilar misfolds is necessary and sufficient for a sequence to be protein-like, i.e. to be stable and to fold rapidly into its native conformation. Mean-field theory of protein folding predicts that one of the lowest energy in the ensemble of misfolded conformations depends on amino acid composition only [6, 7], while the energy of the native state crucially depends on sequence. Thus large extensive (in number of amino acid residues) energy gap would guarantee thermodynamic stability and fast cooperative folding for a sequence into its native (lowest energy) conformation. Accordingly, the definition of designability of a structure was generalized in [8, 9] as the number of sequences that can fold into that structure with large energy gaps. The cumulative sequence entropy curve (SEC), defined as the logarithm of the number of sequences that can fold into a given conformation with energy equal or lower than E, was derived for several target protein structures in [8]. The important question of structural determinant of protein designability, i.e. which features of the target structure determine the SEC for this structure, was addressed in [9]. The authors of [9] argued that the maximal eigenvalue of the contact matrix can serve as a good predictor of SEC, i.e. of the designability of a structure. Shakhnovich et al presented an intuitive explanation for this result in [10] by relating the maximal eigenvalue of contact matrix to the number of closed loops passing through the system of intramolecular inter-amino acid contacts and analyzing their contributions to the energy of a sequence in the native structure. It was also shown that the structures of proteins from thermophilic organisms are more designable [11] than the ones from their mesophilic counterparts. Furthermore structures of proteins believed to belong to last universal common ancestor (LUCA) appear to be highly designable, perhaps reflecting the thermophilic nature of earlier life.

The largest eigenvalue of the contact maps c_{ij} are listed in Table 1 of the main text for the four structures S_A, S_B, S_C, S_D. We stress that, in light of the results for the pressures λ_{ij} obtained in the present paper, estimates of designability should depend on $\mathbf{c} - \bar{\mathbf{c}}$ and not $\mathbf{c}$ alone due to the effects of competitor folds onto the designability. Inverse statistical modeling taking into account the covariation between amino acids offer more accurate estimates of the designability. In particular, the ACE algorithm provides an approximation of the entropy of the inferred Potts model [12], in addition to the values of the inferred couplings. In Table 1 of the main text we list the values of the entropies of the Potts models inferred for the four structures considered here. The Potts entropy is bounded from above by $27 \times \log 20 \simeq 80.9$; the difference between this upper bound (corresponding to a set of $L = 27$ fully unconstrained amino acids) and the Potts entropy is a measure of the structural constraints acting on the sequences. A recent, detailed study of the notion of entropy of protein families, with application to real and lattice data can be found in [12].

II. IMPLEMENTATION OF THE INFERENCE PROCEDURE AND VALIDATION

Let $L = 27$ be the length of the proteins, and $q = 20$ the number of amino acids. One- and two-point frequencies measured from the data provide $Lq + L(L-1)/2q^2$ data points to be fitted within the Potts model (see Eq.(6) and Eq.(7) in Methods Section) with the same number of field and coupling parameters. Since $\sum_{a=1}^{q} f_i(a) = 1 \;\; \forall i$, L, free parameters are removed. In addition, $\sum_{a=1}^{q} f_{ij}(a,b) = f_j(b)$ for all j, b and $\sum_{b=1}^{q} f_{ij}(a,b) = f_i(a)$ for all i, a. Those equalities remove another set of $\frac{1}{2}L(L-1) \times (2q-1)$ free parameters. The inference is thus done by considering fields and couplings that depend on $q-1$ symbols (amino-acid values) only. We are free to choose, for each site, which symbol is removed. In the main paper, we show results corresponding to the consensus gauge, where the amino acid with maximum frequency is removed, or the least-probable gauge, where the amino acid with minimum frequency is removed. Couplings may also be expressed in the zero-sum gauge, where the sums over each row or column of the coupling matrix (attached to any pair of sites i, j) are equal to zero.

A. Independent-site Model (IM)

In the Independent-site Model (IM) framework we infer a Potts model that reproduces the amino-acid frequencies on all sites, without caring about correlations. The Independent-site Potts Model is fully determined by its fields,

$$h_i^{\mathrm{IM}}(a) = \log\left(\frac{f_i(a)}{f_i(a_i)}\right) \;, \tag{1}$$

where a_i is the index of the consensus amino acid at site i. We therefore work in the consensus gauge: on each site i we remove the symbol a_i from the list of amino acids. Amino acids a that have not been observed in the MSA at a given site i are also discarded; the numbers of amino acids retained therefore vary from site to site.

B. Mean-Field and Gaussian Models (DCA)

The coupling matrix inferred according to the Mean-Field DCA approach [13] is simply the opposite of the inverse of the pairwise covariance matrix between residues. In our MSA data (as in the case of real proteins), some amino-acids may never be observed on some sites of the protein. In that case the two-point frequency matrix acquire zero modes, which makes the inversion procedure ill-defined. This problem can be cured by resorting to pseudo-counts, and we use this standard method when applying DCA; the introduction of large pseudo- counts is also useful to cure the errors introduced by the mean-field approximation [14].

More precisely, we compute the pairwise correlations $f_{ij}(a,b)$ and frequencies $f_i(a)$ from the MSA. Note that, to remove sampling biases producing correlations between the different sequences in real MSA, a reweighting of the sequences is usually performed in the computation of those statistical quantities, according to their distances with the other sequences in the MSA (Methods, main text). In practice, we have not considered any reweighing here, except in the case of biased sampling with a penalty term discussing sequences with low identity with respect to a reference sequence (Methods, man text).

We then introduce a pseudo-count α by transforming the 1- and 2-site statistics in the following way:

$$f_i(a) \to f_i^{\mathrm{PC}}(a) = (1-\alpha) f_i(a) + \frac{\alpha}{q}$$
$$f_{ij}(a,b) \to f_{ij}^{\mathrm{PC}}(a,b) = (1-\alpha) f_{ij}(a,b) + \frac{\alpha}{q^2} \tag{2}$$

Here we use the value $\alpha = 0.5$ that is used when considering protein data, but we note that this value could be in principle optimized for the case of lattice proteins. Once the pseudocounts have been taken into account, the DCA couplings are given by the inverse of the connected correlation matrix $\boldsymbol{c}$ defined by

$$c_{ij}(a,b) = f_{ij}^{\mathrm{PC}}(a,b) - f_i^{\mathrm{PC}}(a) f_j^{\mathrm{PC}}(b) \tag{3}$$

However, the identities $\sum_b f_{ij}^{\mathrm{PC}}(a,b) = f_i^{\mathrm{PC}}(a)$ give a trivial zero-mode to the $\boldsymbol{c}$ matrix, that is removed by using the 'consensus gauge' as discussed above for the IM case. Removing the row a_i and column a_j of the matrix $\boldsymbol{c}$ for every pair i, j, we obtain the correlation matrix $\boldsymbol{c}^{\mathrm{cons}}$ in the consensus gauge. The couplings are given by

$$J_{ij}^{\mathrm{DCA}}(a,b) = -\left(\boldsymbol{c}^{\mathrm{cons}}\right)^{-1}_{ij}(a,b) \tag{4}$$

and the couplings for the consensus amino acids are set to zero by definition.

The coupling matrix inferred in the DCA approach above defines a Gaussian model for the sequences $\boldsymbol{A} = (a_1, a_2, \ldots, a_L)$, with probability distribution

$$P^G(\boldsymbol{A}) \propto \exp\left[\frac{1}{2}\sum_{i,j}\sum_{a,b} J_{ij}^G(a,b)\,\big(\sigma_{i,a}(\boldsymbol{A}) - f_i(a)\big)\,\big(\sigma_{j,b}(\boldsymbol{A}) - f_j(b)\big)\right] , \tag{5}$$

up to a multiplicative normalization constant. In the formula above, the couplings are equal to their DCA values: $J_{ij}^G(a,b) = J_{ij}^{DCA}(a,b)$. Variable $\sigma_{i,a}(\boldsymbol{A})$ is equal to 1 if $a = a_i$, and to 0 otherwise. Note that the discrete nature of the $\sigma_{i,a}$–variables make P^G approximate. Within this Gaussian approximation the fields read

$$h_i^G(a) = -\sum_{i\neq j,b} J_{ij}^G(a,b)\, f_j(b) - \sum_{b\neq a} J_{ii}^G(a,b)\, f_i(b) + \frac{1}{2} J_{ii}^G(a,a)\,(1 - 2\, f_i(a)) \ . \tag{6}$$

Given those field and couplings values, one can sample the Gaussian distribution to generate new sequences, based on Eq. [9] in the main text, with the results shown in Fig. 3A in the main text.

C. Pseudo-likelihood Method (PLM)

To infer the Potts couplings and fields with the Pseudo-Likelihood Method we have used the asymmetric Pseudo-likelihood code of [15], with regularization strength $\gamma = 0.01$. The inferred couplings are then transformed in the

zero-sum gauge, *i.e.* the sums over all rows and columns of $J_{ij}(a,b)$ vanish. This gauge is empirically known to give the best performance for contact predictions based on PLM couplings. We have indeed verified that contact prediction based on PLM-couplings in the consensus gauge is less accurate.

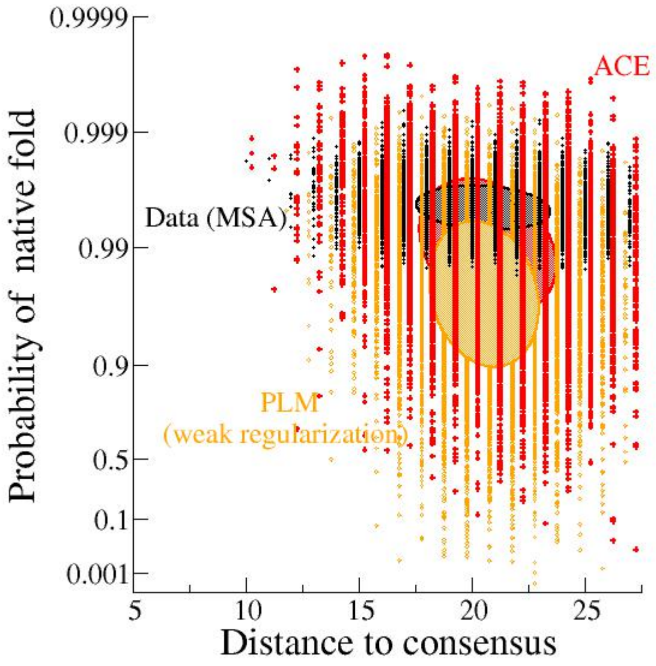

FIG. B: Same as Fig. 3A of the main text, but for the weakly regularised Potts-PLM model ($\gamma = 0.0001$) instead of the strongly regularized Potts-PLM model ($\gamma = 0.01$) usually considered for structural predictions. Gaussian and IM data are not shown.

While PLM with large regularization strength is known to provide optimal contact prediction [15], we have also inferred the PLM couplings with weaker regularisation strength $\gamma = 0.0001$. As shown in Fig. B, sequences generated through MC sampling of the corresponding Potts-PLM model have higher probabilities of folding in the native structure than sequences sampled from the strongly regularised Potts-PLM model, compare to Fig. 3A in the main text.

D. Adaptive Cluster Expansion (ACE)

For the ACE-based inference of [16, 17] adapted to Potts variables [18], no pseudo-count is required but only amino acids with non-zero frequencies are retained as Potts symbols in the inferred model. The number of Potts symbols present on each site is therefore a variable parameter along the protein sequence. The inference is done in the least-probable gauge, in which the fields parameters of the least frequent amino acid in each site and each coupling parameter with him are set to zero. A L_2-regularization, with small strength $\gamma = 0.0001 = 5/M$, where $M = 5\ 10^4$ is the number of sequences in the MSA, is used in the inference.

In the ACE method, clusters of interacting sites are gradually incorporated if their contributions to the cross-entropy exceed some threshold t. The cross-entropy converges to a plateau value, as t is lowered and clusters with smaller and smaller contributions are taken into account, see Fig. C. The inference procedure requires to compute the partition function of the Potts model restricted to those clusters. Such a computation becomes prohibitively slow when cluster include more than, say, $K_{max} = 7$ sites each carrying $q \simeq 20$ Potts states. We have at this point stopped the ACE-inference, as shown by the threshold indicated in Fig. C. We have then refined the inferred couplings and field parameters with a Boltzmann machine learning (BML) procedure, to reproduce the 1- and 2-site statistics within the expected fluctuations due to the finite sampling. The output of the ACE algorithm provides a good guess of the couplings and fields, and BML is fast. As shown in Fig. C, structures S_C and S_D give rise to smaller thresholds and to faster convergence of the inference procedure, with respect to S_A and S_B. The latter structures are indeed less designable (Section III.B). In Fig. D we show the histograms of the inferred couplings (with ACE) for the four structures. The couplings inferred for structures S_A and S_B are slightly larger than their counterparts for the other two structures, in particular, S_C.

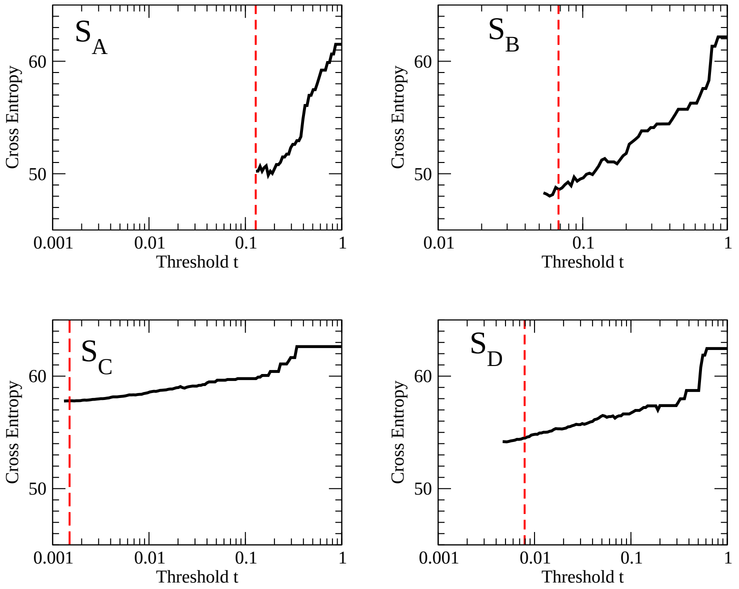

FIG. C: Evolution of the estimated entropy of the Potts model inferred from the MSAs attached to the four structures under study along the evolution of the ACE inference procedure. The entropy has stabilized when the cut-off over the contribution to the entropy coming from clusters (threshold) becomes small, indicating that the inference is successful. Note that the final values of the entropy shown in the figure exceed by about 0.5 the values reported in Table 1 of the main paper, due to the contribution of the regularization terms, see [12] for a detailed discussion.

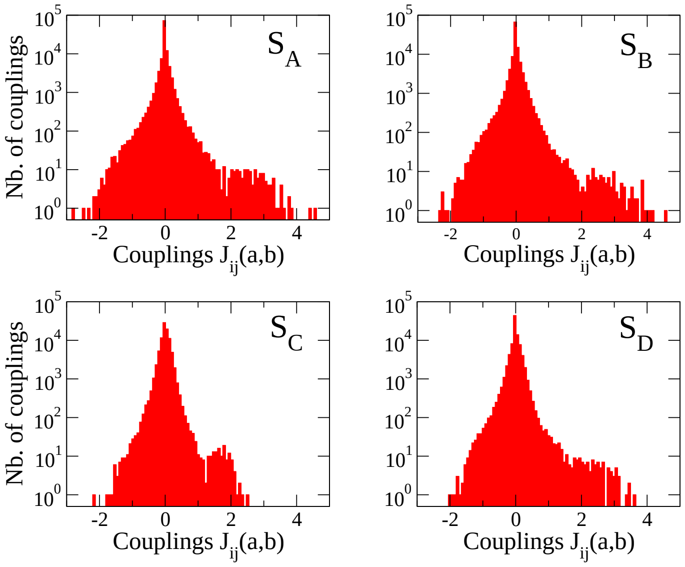

FIG. D: Histograms of the inferred Potts-ACE couplings for the four structures S_A, S_B, S_C and S_D.

To explicitly check that the inference was successful, we compare in Fig. E the one- and two-point statistics computed from a large number of sequences generated from the inferred Potts model with a Monte Carlo procedure to their values in the MSA of structure S_B. We see that the single-site frequencies $f_i(a)$ and the two-point connected correlations $c_{ij}(a,b)$ (Methods in main paper) are perfectly reproduced.

We can also perform more detailed comparisons to show that the model is indeed predictive, beyond one- and two-point functions. For example the connected three-point correlations

$$c_{ijk}(a,b,c) = \langle \delta_{a_i,a}\delta_{a_j,b}\delta_{a_k,c} \rangle - f_i(a)f_{jk}(b,c) - f_j(b)f_{ik}(a,c) - f_k(c)f_{ij}(a,b) + 2f_i(a)f_j(b)f_k(c) \tag{7}$$

computed from the data are in very good agreement with their counterparts computed from the inferred Potts model (with Monte Carlo sampling), see right panel of Fig. E.

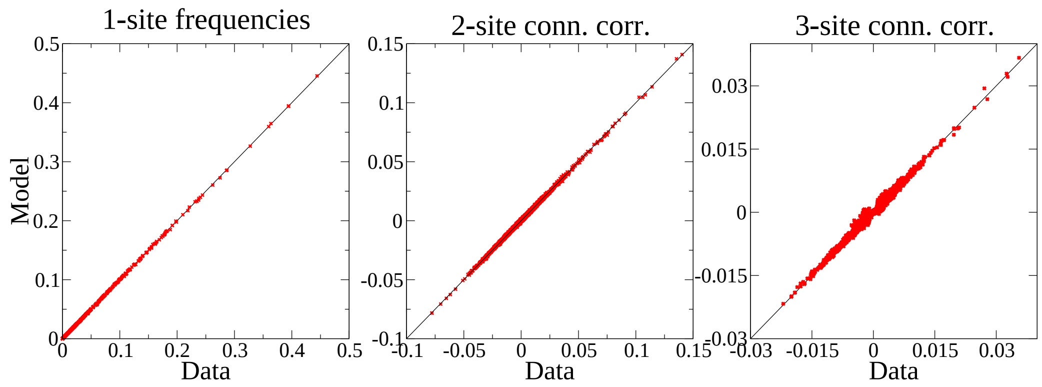

FIG. E: Comparison of amino-acid statistics in the MSA of structure S_B (x–axis) and in the corresponding inferred Potts-ACE model (y–axis). The one-point frequencies $f_i(a)$ and the two-point connected correlations $c_{ij}(a,b) = f_{ij}(a,b) - f_i(a)f_j(b)$ are shown, respectively, in the left and middle panels. The straight line $x = y$ corresponds to perfect matching. The inference problem is accurately solved. The MSA was made of sequences were sampled at temperature $\beta = 10^3$, in the presence of a reduced pool of 10^4 randomly chosen structures. The three-point connected correlation functions, defined in Eq. (7), are shown in the right panel; the coefficient of determination is equal to $R^2 \simeq 0.98$.

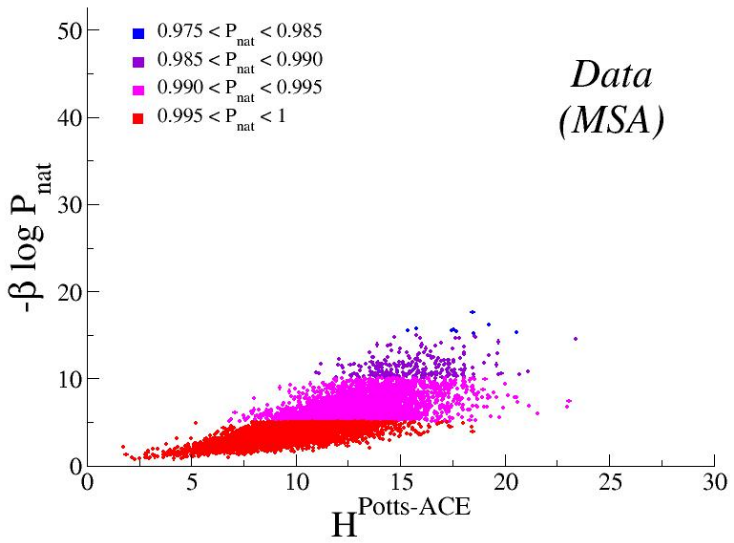

FIG. F: Scatter plot of the 'energy' $\mathcal{H}^{Potts-ACE}[\mathbf{A};\mathbf{h},\mathbf{J}]$, Eq. [9] in the main text, with the inferred Potts-ACE (x-axis) vs. effective Hamiltonian $\beta\mathcal{H}^{\rm LP}[\mathbf{A}|S] = -\beta \log P_{nat}(S|\mathbf{A})$, Eq. [7] in the main text (y-axis), for 10,000 sequences in the 'natural' MSA generated for structure S_B (at inverse sampling temperature $\beta = 1000$). Colors identify intervals of values for P_{nat}, see legend in panel. The energy of the sequences computed with the Potts-ACE model have been subtracted the energy of the best folder, such that the minimal energy is zero.

We report in Fig. F the scatter plot of the 'energies' of the sequences in the 'natural' MSA, computed with the inferred Potts-ACE model and with the exact LP Hamiltonian, $-\beta \log P_{nat}$, see Methods in the main text. We observe that the energies are strongly correlated: the lower the Potts-ACE energy, the higher the value of the probability of folding, P_{nat}. The scatter plot for natural sequences is quantitatively similar to the low-energy part of the scatter plot corresponding to the Potts-ACE generated sequences, see Fig. 3B in the main text.

III. CALCULATION OF THE PRESSURE λ^{TH}

The effective Hamiltonian of a sequence $\mathbf{A}$ folding in structure S_{nat} in the Monte Carlo simulations (see Methods section on the sampling of the sequence space in the main paper) reads

$$\mathcal{H}(\boldsymbol{A}) \equiv -\beta \ln P_{nat}(\mathbf{A}) = \beta \ln \left(1 + \sum_{S(\neq S_{nat})} \exp \left(\sum_{i<j} \left[c_{ij}^{(S_{nat})} - c_{ij}^{(S)} \right] E(a_i, a_j) \right) \right) \ . \tag{8}$$

Note that the form of $\mathcal{H}$ implies effective multi-body terms that originate from the need to avoid competing folds. We now derive the effective pairwise Potts Hamiltonian which approximates $\mathcal{H}$ in the case of large inverse temperatures β. For the sake of simplicity, we first consider the limiting case of two structures only in Section III A. The general case of multiple structures in competition is then studied in Section III B.

A. Case of two structures

Let us assume first that we have only the native structure, S_{nat}, with contact matrix $c_{ij}^{(S_{nat})}$, and another 'competing' fold, S_{comp}, with contact map $c_{ij}^{(S_{comp})}$. Let us define the difference between the two contact maps

$$\Delta c_{ij} = c_{ij}^{(S_{nat})} - c_{ij}^{(S_{comp})} \ . \tag{9}$$

For each pair of residues i, j, Δc_{ij} is equal to $+1$ if i, j are in contact on the native fold but not on its competitor, 0 if they are in contact or not in contact on both folds, and -1 if they are in contact on the competitor but on the native fold. The effective Hamiltonian (8) is $\mathcal{H}(\boldsymbol{A}) = \beta \ln \left(1 + e^{-G(\boldsymbol{A})}\right)$ where the 'gap' function is

$$G(\boldsymbol{A}) \equiv -\sum_{i<j} \Delta c_{ij} \, E(a_i, a_j) \ . \tag{10}$$

The sequences $\boldsymbol{A}$ with very high probabilities of folding into S_{nat} are the sequences with very large and positive gaps $G(\boldsymbol{A})$. Let us call $\boldsymbol{A}^{GS}$ the ground state of $\mathcal{H}$, *i.e.* the sequence maximizing the gap G. The sequences $\boldsymbol{A}$ belonging to the MSA after generation by the Monte Carlo procedure correspond to slightly larger value for the Hamiltonian $\mathcal{H}$, with $\mathcal{H}(\boldsymbol{A}) - \mathcal{H}(\boldsymbol{A}^{GS})$ typically less than or equal to unity. Therefore:

$$\mathcal{H}(\boldsymbol{A}) - \mathcal{H}(\boldsymbol{A}^{GS}) = \beta \ln \left(\frac{1 + e^{-G(\boldsymbol{A})}}{1 + e^{-G(\boldsymbol{A}^{GS})}} \right) \leq 1 \ , \tag{11}$$

Using the fact that both gaps $G(\boldsymbol{A})$ and $G(\boldsymbol{A}^{GS})$ are large, we obtain after expansion of the logarithm to the first order,

$$\mathcal{H}(\boldsymbol{A}) - \mathcal{H}(\boldsymbol{A}^{GS}) \approx \beta \, \left(e^{-G(\boldsymbol{A})} - e^{-G(\boldsymbol{A}^{GS})} \right) = \beta \, e^{-G(\boldsymbol{A}^{GS})} \, \left(e^{G(\boldsymbol{A}^{GS}) - G(\boldsymbol{A})} - 1 \right) \leq 1 \ , \tag{12}$$

or, equivalently, that the gap associated to sequence $\boldsymbol{A}$ is below its maximal value $\boldsymbol{A}^{GS}$ by at most

$$\delta G_{max} \equiv \ln \left(\frac{1}{\beta \, e^{-G(\boldsymbol{A}^{GS})}} + 1 \right) . \tag{13}$$

In physical terms, we interpret the result above as if sequences $\boldsymbol{A}$, whose true associated energy is $\mathcal{H}$ and whose true temperature is equal to unity, were distributed according to the Gibbs distribution associated to the energy $-G(\boldsymbol{A})$ at the (very low) effective temperature δG_{max}. For sequences in the MSA with large folding probabilities we can therefore approximate the Hamiltonian (in units of the effective temperature) as

$$\frac{-G(\boldsymbol{A})}{\delta G_{max}} = \frac{1}{\delta G_{max}} \sum_{i<j} \Delta c_{ij} \; E(a_i, a_j) \ . \tag{14}$$

Hence, the Hamiltonian $\mathcal{H}(\boldsymbol{A})$ can be approximated with a Potts model with pairwise interactions given by

$$J_{ij}(a,b) = -\frac{\Delta c_{ij}}{\delta G_{max}} \; E(a,b) \ . \tag{15}$$

We obtain the value of the pressure λ_{ij}^{TH} announced in Eq. [3] of the main text, in the special case $N_S = 1$, $\Delta = G(\boldsymbol{A}^{GS})$, and $\bar{c}_{ij} = c_{ij}^{(S_{comp})}$.

B. Case of multiple structures

We now turn to the generic case of multiple structures S competing with the native fold S_{nat}. As explained in the Methods section of the main paper, we define the gap $\Delta(S|S_{nat})$ between the competing structure S and the native fold S_{nat} as the average value of the ratio of the Boltzmann weights of the sequences $\boldsymbol{A}$ in the MSA of S_{nat} to fold, respectively, in structures S and S_{nat}. The weight of the structure S is then defined through

$$\mu(S) = e^{-\Delta(S|S_{nat})} \ . \tag{16}$$

The 100 top weights of the structures competing with $S_{nat} = S_A, S_B, S_C, S_D$ are shown in decreasing order in Fig. G. We observe that only a small number N_S of structures S have large weights μ; this number M is variable from one native structure to another. The number M of competitor structures and their typical gap Δ with the native structure are approximated from the sum of the weights $\mu(S)$ over all the structures S different from S_{nat}, see Methods in the main paper. Furthermore, we define the average contact map $\bar{c}_{ij}$ of those competitors as the average value of the contact maps $c_{ij}^{(S)}$ over all the structures S different from S_{nat}, weighted by $\mu(S)$, see Methods in the main paper. Note that the sum of $\bar{c}_{ij}$ over all pairs $i < j$ is equal to 28, and each entry of this matrix ranges between 0 and 1.

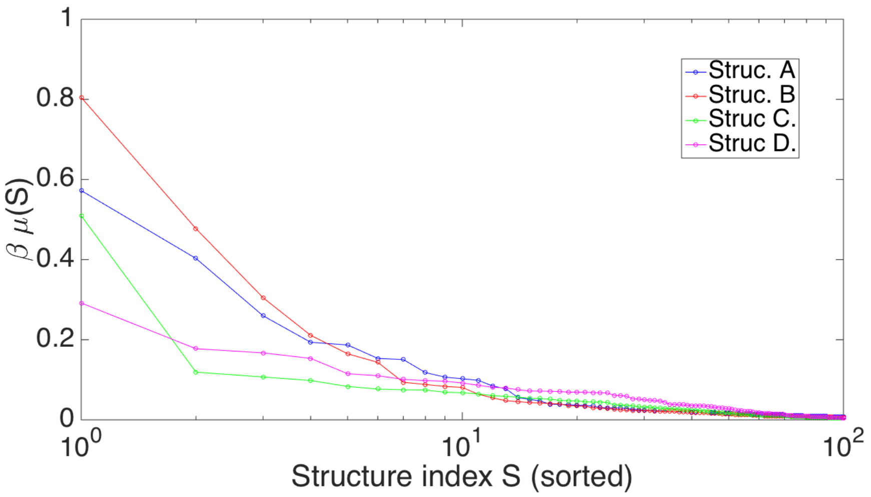

FIG. G: Weights $\mu(S)$, multiplied by the inverse sampling temperature $\beta = 10^3$, of the structures S competing with $S_{nat} = S_A, S_B, S_C, S_D$, and sorted in decreasing order.

Based on those definitions, we replace the sum over all possible structures (distinct from the native one) in (8) with the sum over the N_S most competing structures, as if they all had the same contact map $\bar{c}$ defined above:

$$\mathcal{H}_{eff}(\boldsymbol{A}) \simeq \beta \ln \left(1 + N_S \ \exp \left[\sum_{i<j} \left(c_{ij}^{(S_{nat})} - \bar{c}_{ij} \right) E(a_i, a_j) \right] \right) \ . \tag{17}$$

This expression makes sense only for sequences $\boldsymbol{A}$ that are very good native folders. Within this approximation, we recover the 'gap' function $G(\boldsymbol{A})$ of Eq. (10), where the 'differential' contact map is $\Delta c_{ij} = c_{ij}^{(S_{nat})} - \bar{c}_{ij}$ and with the additive constant $-\ln N_S$. The calculation of Section III A can be repeated, and we readily obtain that the effective temperature δG_{max} is given by (13) with $e^{-G(\boldsymbol{A}^{GS})} = N_S\, e^{-\Delta}$. Hence, the theoretical expression for the local pressure, λ_{ij}^{TH}, given in Eq. [3] of the main text. Applications to the various sampling temperatures and pools of competing structures of Section III.C are reported in Table A.

C. Numerical results for structures S_A, S_C, and S_D

We show the comparison between the inferred and the energetic couplings for structures S_A, S_C, and S_D in, respectively Figs. H, I, and J. We also show the pressures for the different classes of contacts, and the contact maps of the native folds and of the competing structures.

D. Dependence of the pressures on MSA sampling

An interesting feature of the lattice-protein model is that we can restrict the pool of competing structures in the partition function of the model (see Eq. [5] in the Methods section of the main paper). In order to test the effect of competitor structures on the inferred couplings described by Eq. [3] of the main paper, we have generated three pools of possible structures:

- $\Omega_{\rm rand}$, which includes 10^3 randomly chosen structures, plus the native one;
- $\Omega_{\rm close}$, made of the native structure and the 999 closest structures, in terms of the number of common contacts;
- $\Omega_{\rm far}$ made of the native structure and the 999 most distant structures.

The inferred couplings are compared to the MJ energetic parameters in Fig. L. We see that the pressure averaged over the pair of contacts in the native fold, λ, is smaller for the pool of structures $\Omega_{\rm far}$ and is maximal for $\Omega_{\rm close}$. This result agrees with the fact that covariation, measured by the effective couplings, increases with the competition between the native structure and the other structures in the pool. We also show in Fig. L the couplings obtained for a low inverse sampling temperature, $\beta = 10^1$ (all the results shown in the main paper were obtained for $\beta = 10^3$). As expected, we observe that the pressures λ decrease, and so do the inferred couplings. As expected, our theoretical prediction for the pressure, Eq. [3] of the main text, is less accurate at low inverse temperatures.

We give the values of the pressures averaged over the pairs of residues in the classes UN, SN and CC (defined according to the closest competitor S_F) and for the different pools of competing sequences and sampling temperatures in Table A.

	**Pressure (inferred) Eq. [2] of main text**	**Factor** $\beta\, N_S\, e^{-\Delta}$	**Pressure (theory) Eq. [3] of main text**
UN	2.8	5.0357	4.46
SN	1.4	5.0357	2.74
CC	-0.9	5.0357	-1.79
$\Omega_{\rm far}$	1.30	1.3208	1.66
$\Omega_{\rm close}$	2.75	5.0411	3.40
$\Omega_{\rm rand}$	2.24	3.4319	2.81
$\beta = 10$	1.94	5.3425	4.02
$\beta = 100$	2.3	4.3598	3.24

TABLE A: Pressures λ averaged over all pairs of sites in contact, or only over the pairs in the classes UN, SN, CC (see Fig. 4 of the main paper, $\beta = 1000$) as we vary the pools of competing structures and the inverse sampling temperature for structure S_B.

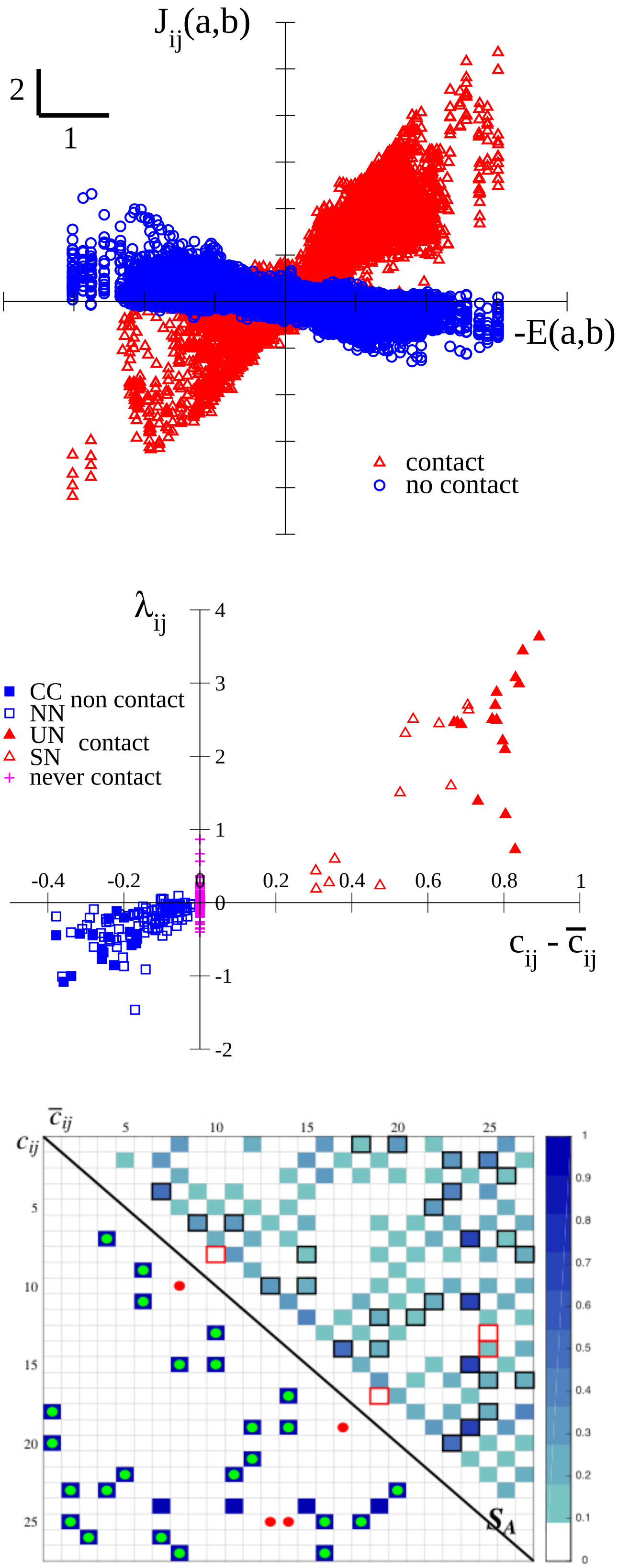

FIG. H: Structure S_A. **Top.** Comparison between inferred and energetic couplings (only one over 50 points are shown); red symbols corresponds to pairs of sites in contact, blue symbols to pairs of sites that are not in contact. **Middle.** Pressures λ_{ij} vs. $c_{ij} - \bar{c}_{ij}$. The classes UN, SN, CC and NN were defined according to the closest competitor structure of S_A, *i.e.* with the smallest effective gap $\Delta(S|S_A)$, denoted by S_G, see Fig. K. **Bottom.** Lower triangle: contact map c_{ij} shown with full blue squares. True and false positives predicted from 28 top F^{APC} scores with the ACE method are shown with, respectively, green and red dots. Upper triangle: contact map $\bar{c}_{ij}$ averaged over all competitor folds with their Boltzmann weights. Missed contacts i, j in the prediction have large $\bar{c}_{ij}$ values, and correspond to the center of the fold, $i = 24$. Red squares locate false positives.

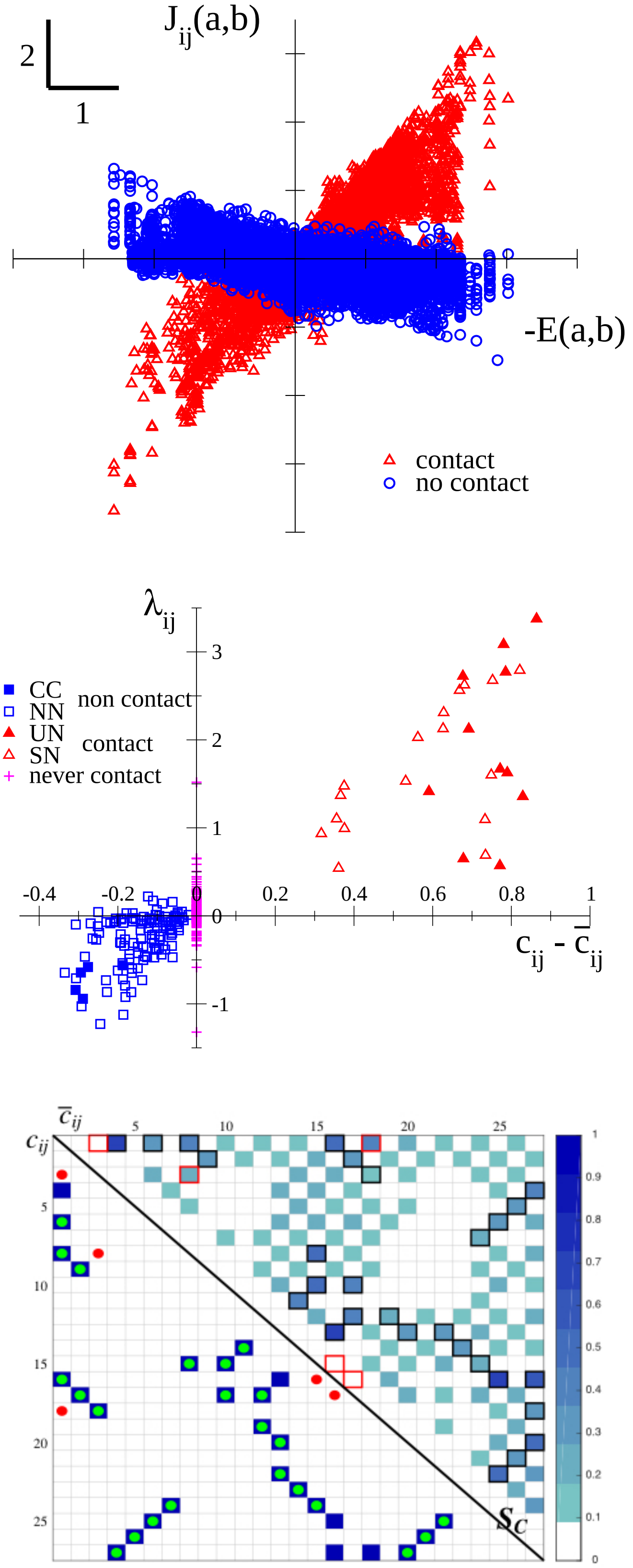

FIG. I: Structure S_C. **Top.** Comparison between inferred and energetic couplings (only one over 50 points are shown); red symbols corresponds to pairs of sites in contact, blue symbols to pairs of sites that are not in contact. **Middle.** Pressures λ_{ij} vs. $c_{ij} - \bar{c}_{ij}$. The classes UN, SN, CC and NN were defined according to the closest competitor structure of S_C, *i.e.* with the smallest effective gap $\Delta(S|S_A)$, structure S_E, see Fig. K. **Bottom.** Lower triangle: contact map c_{ij} shown with full blue squares. True and false positives predicted from 28 top F^{APC} scores with the ACE method are shown with, respectively, green and red dots. Upper triangle: contact map $\bar{c}_{ij}$ averaged over all competitor folds. Red squares locate false positives.

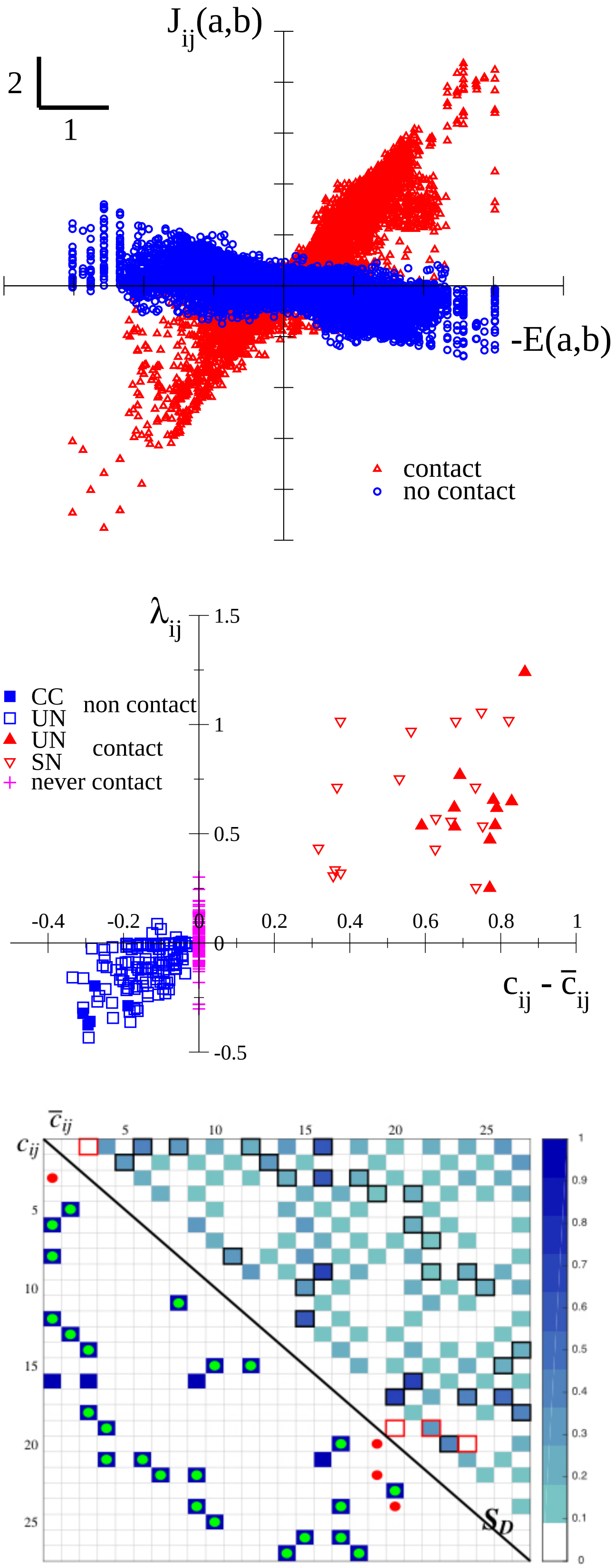

FIG. J: Structure S_D. **Top.** Comparison between inferred and energetic couplings (only one over 50 points are shown); red symbols corresponds to pairs of sites in contact, blue symbols to pairs of sites that are not in contact. **Middle.** Pressures λ_{ij} vs. $c_{ij} - \bar{c_{ij}}$. Structure S_D has several close competitors, see Fig. G; we have thus retained the five structures with the five smallest effective gaps $\Delta(S|S_D)$. We define SN the set of contact pairs i, j such that the average contact map on this 5 structures only is larger than 0.4, the other contacts being pooled into UN. **Bottom.** Lower triangle: contact map c_{ij} shown with full blue squares. True and false positives predicted from 28 top F^{APC} scores with the ACE method are shown with, respectively, green and red dots. Upper triangle: contact map $\bar{c}_{ij}$ averaged over all competitor folds. Red squares locate false positives.

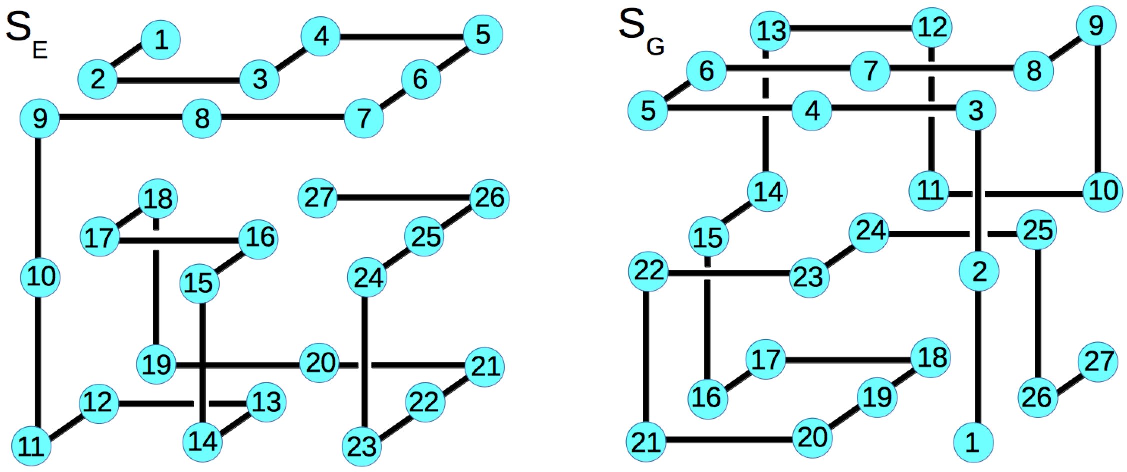

FIG. K: Structures S_E and S_G (from top to down), which are the closest competitors to, respectively, S_C and S_A.

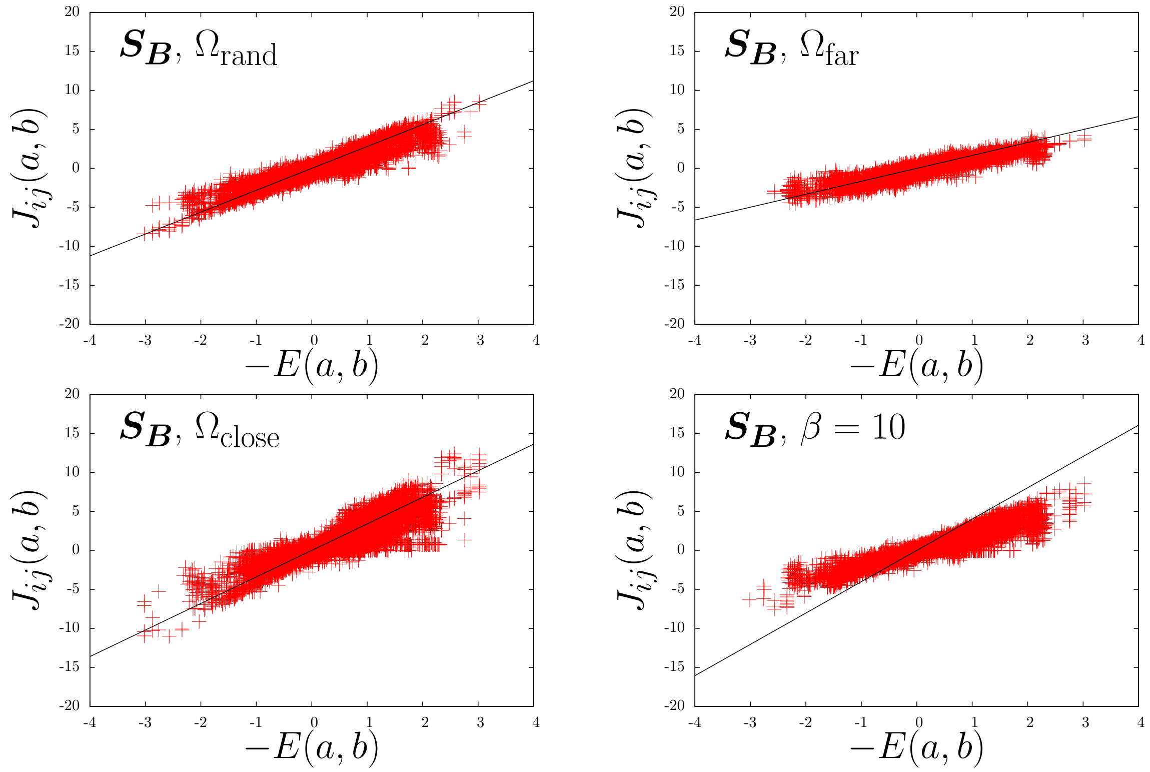

FIG. L: Comparison between inferred and energetic couplings when MSA sampling parameters are varied for structure S_B, either by changing the pool of competing structures or by modifying the sampling temperature. Solid lines are the prediction of the analytical calculation of the average pressure (see Eq. [3] of the main paper), see Table A. Linear fits give slopes of 2.23, 2.76, 1.28 and 1.89 for, respectively, rand, far, close and $\beta = 10$.

IV. CONCENTRATION OF SEQUENCE DISTRIBUTION DUE TO GAUSSIAN INFERENCE

We now illustrate on two very simple examples the biases introduced by the Gaussian distribution P^G (5). For the sake of simplicity, we consider only the case of amino acids taking $q = 2$ values, but our results are qualitatively valid for generic q.

A. Case of a single amino acid

We consider first the case of a single amino acid (sequence of length unity), taking one of two values, say, $a = 0$ with frequency f_0 and $a = 1$ with frequency $f_1 = 1 - f_0$. It is natural to choose the zero-sum gauge, in which the sum over all lines and all columns of the correlation and coupling matrices is zero. The covariance matrix and the coupling matrix (within the mean field approximation) are

$$\hat{c} = f_0(1 - f_0) \times \begin{pmatrix} 1 & -1 \\ -1 & 1 \end{pmatrix} , \quad \hat{J} = \frac{1}{2f_0(1 - f_0)} \times \begin{pmatrix} 1 & -1 \\ -1 & 1 \end{pmatrix} . \tag{18}$$

Inserting this expression in Eq. (5), we obtain the probabilities of the two amino acids within the Mean-Field approximation:

$$P^G(a = 0) = \frac{e^{f_0/(1-f_0)}}{e^{(1-f_0)/f_0} + e^{f_0/(1-f_0)}} \quad \text{and} \quad P^G(a = 1) = \frac{e^{(1-f_0)/f_0}}{e^{(1-f_0)/f_0} + e^{f_0/(1-f_0)}} . \tag{19}$$

We plot the probability of state $a = 0$ as a function of its 'true' value f_0 in Fig. M, left. We observe that the Gaussian approximation recovers correctly the unbiased case ($P^G = \frac{1}{2}$ if $f_0 = \frac{1}{2}$), but strongly overestimates the bias in the true frequency otherwise. In other words, the Gaussian distribution is strongly concentrated around the consensus amino acid, see entropies plotted Fig. M, right.

B. Case of a sequence with two amino acids

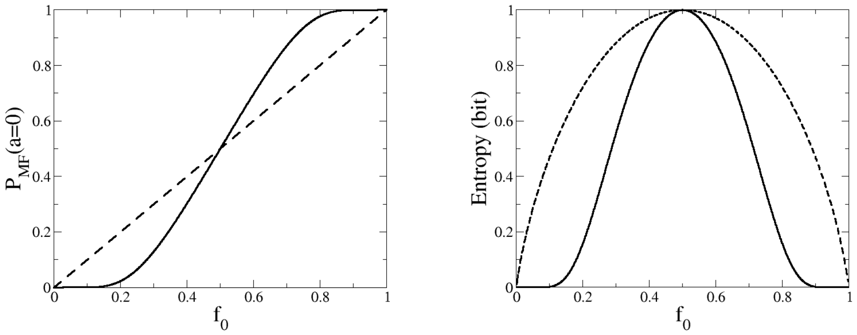

FIG. M: **Left.** The continuous line shows the probability that a single amino acid take one out of two possible states in the Gaussian approximation (y–axis), see Eq. (19) vs. its true value (x–axis). The dashed line is $x = y$. **Right.** Entropies of the Gaussian (full line) and true (dashed line) distributions.

We now consider the case of a two amino-acid sequence, a_1 and a_2, each of which take two possible values, 0 or 1. The probabilities of the four possible configuration of the sequence $\boldsymbol{A} = (a_1, a_2)$ are given by

$$f(0,0) = \frac{1}{Z}, \; f(0,1) = f(1,0) = \frac{e^h}{Z}, \; f(1,1) = \frac{e^{2h+J}}{Z} \quad \text{where} \quad Z = 1 + 2\,e^h + e^{2h+J} . \tag{20}$$

When the coupling J is different from zero the two amino acids covary. The Gaussian distribution can be easily computed for this simple system. We do not reproduce the formulas here, but rather plot the entropy of the Gaussian distribution as a function of the correlation c between the amino-acids value,

$$c = f(1,1) - f(1,0)f(0,1) , \tag{21}$$

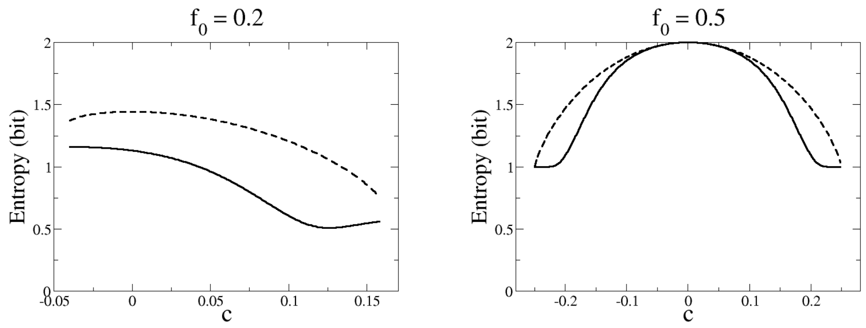

FIG. N: Entropy of the Gaussian (full line) and true (dashed line) distributions for $f_0 = 0.2$ (**left**) and $f_0 = 0.5$ (**right**) vs. correlation c between the amino-acid values, see Eq. (21). The curves end up at the maximal and minimal possible values of c given f_0. For $c = 0$ the two amino acids are uncoupled and the entropies coincides with the values shown in Fig. M, right.

and compare it to the one of the true distribution. Results are shown in Fig. N for two values of $f_0 = f(1,0) = f(0,1)$. Again, as in the single-amino acid case, we find that the entropy of the Gaussian approximation is always smaller than the one of the true distribution. This proves that the Gaussian distribution is more concentrated than the true distribution.

---

[1] I.N. Berezovsky, K.B. Zeldovich, and E. Shakhnovich. Positive and negative design in stability and thermal adaptation of natural proteins. *PLoS Comput Biol*, 3(3):e52, 03 2007.

[2] H. Li, R. Helling, C. Tang, and N. Wingreen. Emergence of preferred structures in a simple model of protein folding. *Science*, 273:666–669, 1996.

[3] E. Shakhnovich and A. Gutin. Enumeration of all compact conformations of copolymers with random sequence of links. *Journal of Chemical Physics*, 93:5967–5971, 1990.

[4] A. Sali, E. Shakhnovich, and M. Karplus. How does a protein fold? *Nature*, 369(6477):248–251, 05 1994.

[5] E. Shakhnovich. Proteins with selected sequences fold into unique native conformation. *Physical Review Letters*, 72:3907–3910, 1994.

[6] E. Shakhnovich. Protein folding thermodynamics and dynamics: Where physics, chemistry, and biology meet. *Chem Rev*, 106:1559–1588, 2006.

[7] E. Shakhnovich and A. Gutin. Engineering of stable and fast-folding sequences of model proteins. *Proc. Natl. Acad. Sci. USA*, 95:7195–7199, 1993.

[8] E. Shakhnovich. Protein design: a perspective from simple tractable models. *Fold Des*, 3:R45–58, 1998.

[9] J.L. England and E.I. Shakhnovich. Structural determinant of protein designability. *Phys. Rev. Lett.*, 90:218101, May 2003.

[10] B.E. Shakhnovich, E. Deeds, C. Delisi, and E. Shakhnovich. Protein structure and evolutionary history determine sequence space topology. *Genome Res.*, 15:385–392, 2005.

[11] J.L. England, B.E. Shakhnovich, and E.I. Shakhnovich. Natural selection of more designable folds: a mechanism for thermophilic adaptation. *Proc. Natl. Acad. Sci. USA*, 100:8727–8731, 2003.

[12] J.P. Barton, A.K. Chakraborty, S. Cocco, H. Jacquin, and R. Monasson. On the entropy of protein families. *Journal of Statistical Physics*, 162:1267-1293, 2016.

[13] F. Morcos, A. Pagnani, B. Lunt, A. Bertolino, D.S. Marks, C. Sander, R. Zecchina, J.N. Onuchic, T. Hwa, and M. Weigt. Direct-coupling analysis of residue coevolution captures native contacts across many protein families. *Proceedings of the National Academy of Sciences of the United States of America*, 108(49):E1293–E1301, 2011.

[14] J.P. Barton, S. Cocco, E. De Leonardis, and R. Monasson. Large pseudocounts and L2-norm penalties are necessary for the mean-field inference of Ising and Potts models. *Physical Review E*, 90(1):012132, July 2014.

[15] M. Ekeberg, E. Hartonen, and E. Aurell. Fast pseudolikelihood maximization for direct-coupling analysis of protein structure from many homologous amino-acid sequences. *J. Comp. Phys.*, 276:341–356, 2014.

[16] S. Cocco and R. Monasson. Adaptive Cluster Expansion for Inferring Boltzmann Machines with Noisy Data. *Physical Review Letters*, 106:090601, 2011.

[17] S. Cocco and R. Monasson. Adaptive Cluster Expansion for the Inverse Ising Problem: Convergence, Algorithm and Tests. *Journal of Statistical Physics*, 147(2):252–314, 2012.

[18] J.P. Barton, E. De Leonardis, A. Coucke, and S. Cocco. ACE: adaptive cluster expansion for maximum entropy graphical model inference http://biorxiv.org/content/early/2016/03/18/044677, 2016.
